# ﻿*Cocconeiscrisscrossis* sp. nov., a new monoraphid diatom (Bacillariophyta) from southern China

**DOI:** 10.3897/phytokeys.242.123316

**Published:** 2024-05-13

**Authors:** Huiwen Zhou, Pan Yu, Luyi Guo, John Patrick Kociolek, Quanxi Wang, Qingmin You

**Affiliations:** 1 College of Life Sciences, Shanghai Normal University, Shanghai 200234, China; 2 College of Environmental and Geographical Sciences, Shanghai Normal University, Shanghai 200234, China; 3 Museum of Natural History and Department of Ecology and Evolutionary Biology, University of Colorado, UCB 218, Boulder CO80309, USA

**Keywords:** diatoms, monoraphid, morphology, new taxa, taxonomy

## Abstract

A novel monoraphid diatom species, *Cocconeiscrisscrossis* You, Yu, Kociolek & Wang, **sp. nov.** is examined and described from the Qingyi River and Maolan Nature Reserve of southern China. The morphological description is based on light microscopy and scanning electron microscopy observations and the new species is compared with similar taxa in this genus. The characteristics unique to *Cocconeiscrisscrossis***sp. nov.** include its central area extending irregularly to both sides, it having closed valvocopulae with heavily silicified fimbriate margins and poles of the valvocopulae have ‘sword-shaped’ siliceous extensions. These features differentiate this new species from others in the genus. This new species was found in alkaline waterbodies, including streams, waterfall and ponds. It was usually found as an epiphyte on the stones; however, it was present on other substrates such as mosses.

## ﻿Introduction

The first described genus of Cocconeidaceae Kützing was *Cocconeis*Ehrenberg (1838). In his early compendium book "*Infusionsthierchen*", Ehrenberg (1838) described the morphology, growth habit and ecology of the genus in French and Latin. The name of the genus is derived from its growth habit, adnate on filamentous algae. *Cocconeis* Ehrenberg is a monoraphid diatom genus widely distributed in marine and freshwater environments, with more than 200 described species, with the main taxa being marine species (Guiry 2023; Kociolek et al. 2023). The frustules of this genus are heterovalvar, with one raphe valve (RV) and one rapheless or sternum valve (SV), where the former is typically less convex than the latter (Jahn et al. 2009; Stancheva 2018; Mora et al. 2022). Solitary cells of *Cocconeis* are attached to filamentous algae or other substrates by their RV, while the SV is exposed to the environment. Valves of *Cocconeis* are elliptical or slightly linear-elliptical in shape and this genus has distinctly different areolae structures on both valves (Round et al. 1990). The areolae of the RV are fine and often have a semicircular hyaline area near the valve ends, while the areolae of the SV are relatively coarse extending to the valve edge and lacking a hyaline area. Striae are usually uniseriate in freshwater species and the poroids are closed by hymenes (Costa et al. 2020). The frustule structure of *Cocconeis* is diverse and complex, species delineation requires detailed observations of the RV morphology, the SV and their connecting elements including valvocopulae and the cingulum (Potapova and Spaulding 2013; Stancheva 2022).

In China, 18 freshwater *Cocconeis* taxa have been recorded, including ten species and eight varieties (Skvortsov 1935; Chin 1951; Zhu and Chen 1989, 1994, 2000; Lin and Wang 1992). China remains a poorly-researched area, with few studies of this genus having gained the attention of researchers. In recent years, we conducted an extensive biodiversity investigation of monoraphid diatoms in China and one new *Cocconeis* species was found, which is distributed in the Qingyi River and Maolan Nature Reserve. This paper describes the frustule morphological characteristics of *C.crisscrossis* sp. nov. using LM and SEM and compares this new species with similar species of the genus.

## ﻿Materials and methods

Diatom samples were collected from two sites: Qingyi River (Yi County, Anhui Province) and Maolan Nature Reserve (Libo County, Guizhou Province). Samples were collected using tweezers or turkey baster. The samples were preserved using formalin solution before being stored in sealed plastic bottles. Sample information is listed in Table 1, including location of samples, longitude and latitude, habitat, pH, conductivity and collection date.

**Table 1.** Locality data and habitat for samples studied.

No. of samples	Location	Coordinates	Habitat	K[μs/cm]	pH	Collection Date
QYJ201710Z12	Qingyi River	30°09′03″N, 117°53′28″E	Attached to stones in the stream	120	8.5	10.1.2017
GZ201510042	Maolan Nature Reserve	25°27'48"N, 107°69'19"E	Attached to stones in the pond	305	7.5	10.2.2015
GZ201510057	Maolan Nature Reserve	25°24'72"N, 107°70'19"E	Attached to floating material near the waterfall	297	7.5	10.2.2015
GZ201510064	Maolan Nature Reserve	25°25'08"N, 107°71'13"E	Attached to stones near the waterfall	297	8.0	10.4.2015
GZ201510088	Maolan Nature Reserve	25°15'71"N, 108°14'22"E	Attached to stones in the stream	296	8.2	10.4.2015
GZ201510096	Maolan Nature Reserve	25°15'74"N, 108°04'18"E	Attached to stones near the waterfall	271	8.2	10.4.2015
GZ201510103	Maolan Nature Reserve	25°17'23"N, 108°04'26"E	Attached to mosses in the stream	272	7.8	10.4.2015

In the laboratory, the samples were processed with concentrated nitric acid using the Microwave Accelerated Reaction System (Model MARS, CEM Corporation, Charlotte, USA); the specific processing and observation steps are described in You et al. (2021). Images were compiled with Adobe Photoshop 2023. Morphological terminology follows Donadel et al. (2018) and Riaux-Gobin et al. (2021). The holotype image of the single specimen is a circled specimen on the type slide. The samples and permanent slides are preserved in Lab of Algae and Environment, College of Life Sciences, Shanghai Normal University.

## ﻿Results

### ﻿ Cocconeis crisscrossis

Taxon classificationPlantaeCocconeidalesCocconeidaceae

Q.M. You, P. Yu, J.P. Kociolek & Q.X. Wang sp. nov.

E3CA1500-D0B6-59B8-9971-A3F484A4F18A

**Figure 1.**
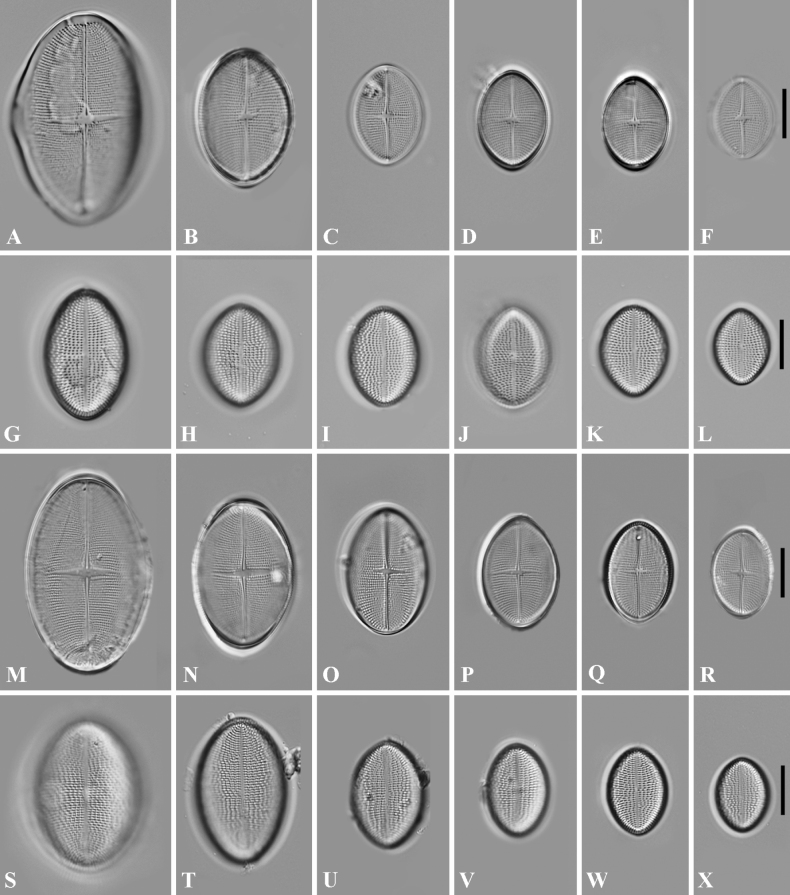
**A–L** Light micrographs of *Cocconeiscrisscrossis* sp. nov. from Qingyi River **M–X** light micrographs of *C.crisscrossis* sp. nov. from Maolan Nature Reserve **A–F, M–R** raphe valves(RV) **G–L, S–X** sternum valves (SV). Scale bars: 10 µm (**A–X**).

**Figure 2.**
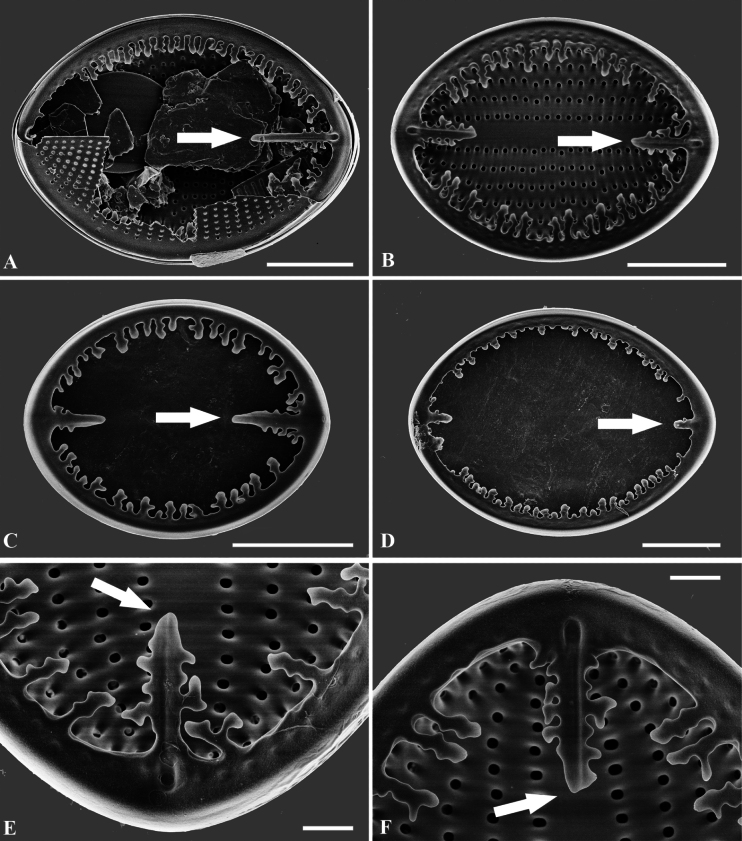
**A–F**SEM micrographs, the valvocopula of *C.crisscrossis* sp. nov. from Qingyi River with well-developed digitate fimbriae, which is close and heavily silicified clamp-like fimbriae seem to be attached to the hyaline rim and are irregularly spaced; the poles of valvocopulae possess unique ‘sword-shaped’ siliceous extensions (arrows in **A–F**) **A** valvocopula of the RV**B–F** valvocopula of the SV. Scale bars: 5 µm (**A–D**); 1 µm (**E, F**).

**Figure 3.**
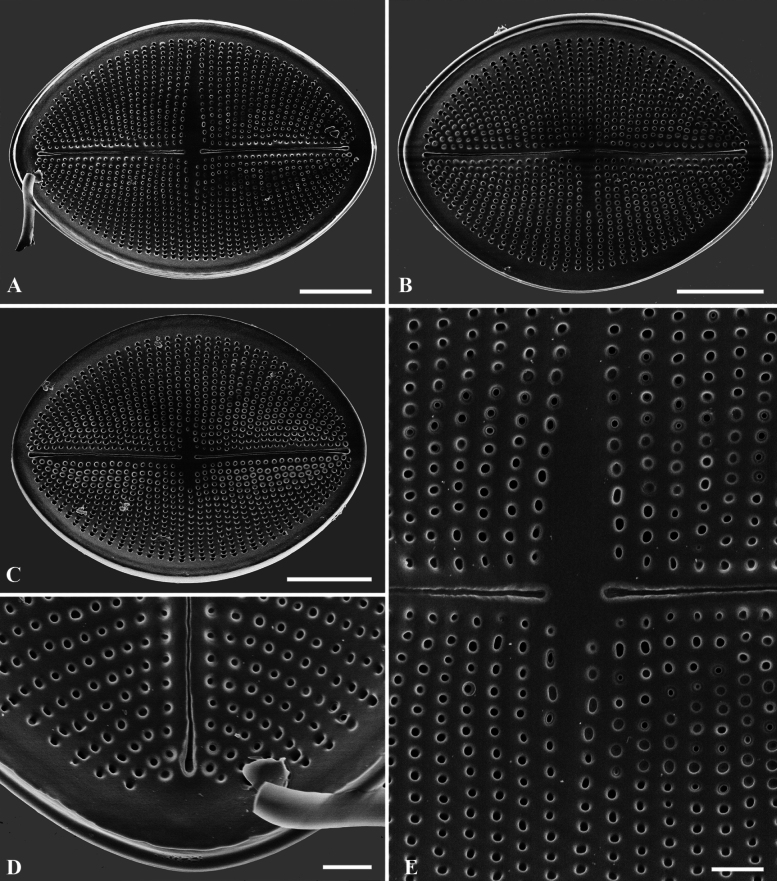
**A–E**SEM micrographs, RV external views of *C.crisscrossis* sp. nov. from Qingyi River **A–C** complete internal valve **D** proximal raphe endings are slightly drop-like expanded **E** irregular hyaline central area. Scale bars: 5 µm (**A–C**); 1 µm (**D, E**).

**Figure 4.**
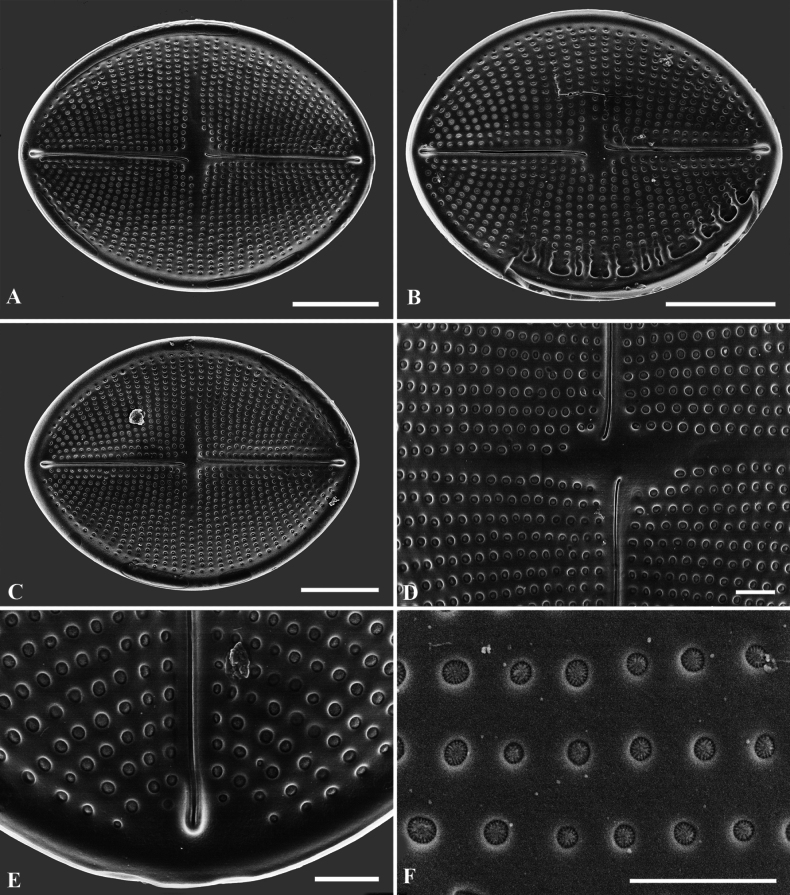
**A–F**SEM micrographs, RV internal views of *C.crisscrossis* sp. nov. from Qingyi River **A–C** complete internal valve **D** irregular hyaline central area and the proximal raphe fissures are bent to opposite sides **E** weakly elevated helictoglossae **F** the areolae are occluded by round lattice-structured hymens. Scale bars: 5 µm (**A–C**); 1 µm (**D–F**).

**Figure 5.**
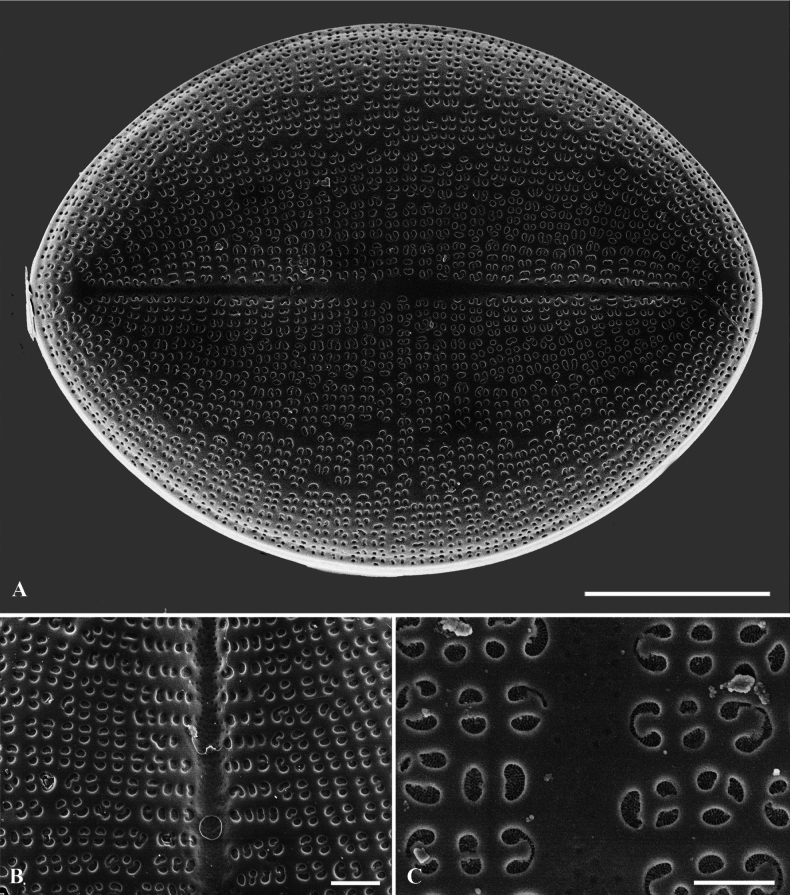
**A–C**SEM micrographs, SV external views of *C.crisscrossis* sp. nov. from Qingyi River **A** complete internal valve **B** narrow and straight sternum **C** Irregular areolae are occluded by lattice-structured hymens. Scale bars: 5 µm (**A**); 1 µm (**B**); 0.5 µm (**C**).

**Figure 6.**
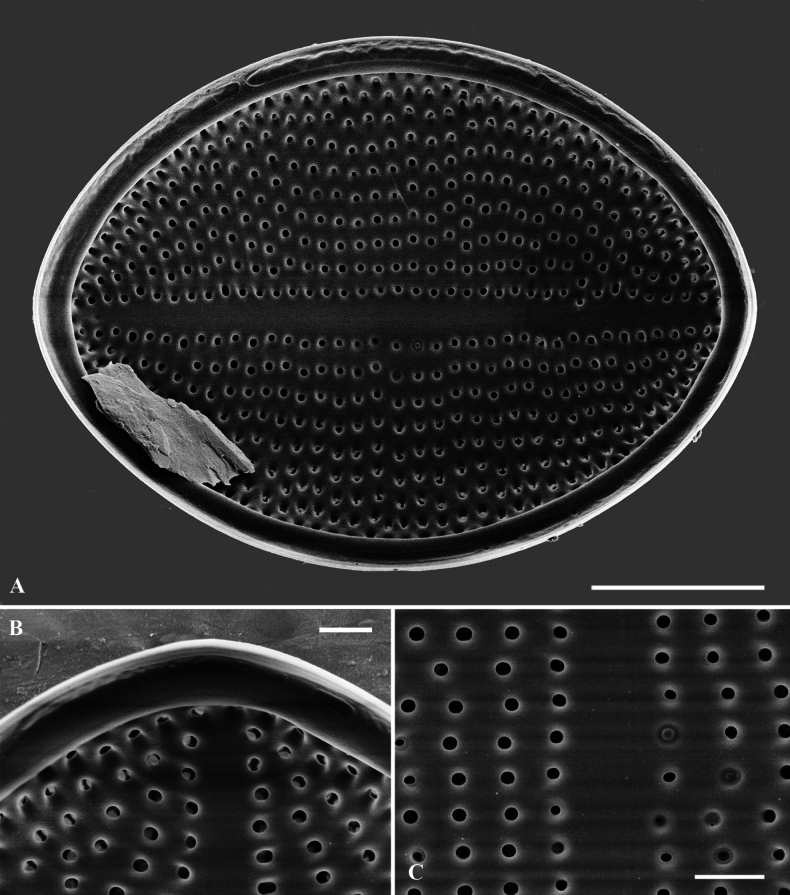
**A–C**SEM micrographs, SV internal views of *C.crisscrossis* sp. nov. from Qingyi River **A** complete internal valve **B, C** detail of the internal valves ends. Scale bars: 5 µm (**A**); 1 µm (**B, C**)).

Figs 1A–X , 2A–F , 3A–E , 4A–F , 5A–C , 6A–C

#### Holotype

**(designated here).** SHTU! slide QYJ201710Z12, holotype illustrated in Fig. 1D, J. Diatom samples are housed in the Lab of Algae and Environment, College of Life Sciences, Shanghai Normal University, China.

#### Type locality.

China. Qingyi River, Anhui Province, 30°9′3″N, 117°53′28″E, collected by Q.X. Wang & P. Yu, 1 October 2017.

#### Description.

Light microscopy (LM) (Fig. 1A–X). Valve elliptical to slight round-elliptical, apices are obtusely rounded; 12.5–42.0 µm long, 9.5–25.0 µm wide. The number of striae on both valves is similar, with parallel orientation at the centre and radiate towards the apices. Both valves display striae with a density of 18–22 rows per 10 µm. Raphe valves (RV) have a thickened hyaline rim on the margin and striae composed of small, nearly round areolae. The raphe is straight, the axial area is narrow, the central area is irregularly narrow, cuniform and the axial area and central area form an irregular cross-like structure. Sternum valve (SV) areolae are rounded and form 7–9 undulating longitudinal lines per hemivalve (increasing in number with increased valve width). The sternum is narrow, linear. The valvocopula has irregularly-spaced digitate fimbriae.

Scanning electron microscopy (SEM) (Figs 2–6) shows RV face is flat with a weakly concave mantle and very narrow linear axial area; the central area extends irregularly to both sides (Figs 3A–C, E, 4A–D). Externally, the raphe is straight, filiform and proximal raphe endings are slightly drop-like and expanded (Fig. 3E). Internally, the proximal raphe fissures are bent to opposite sides (Fig. 4D); the distal raphe endings are straight and end in weakly-elevated helictoglossae on the hyaline rim (Fig. 4E). Striae are made up of round to slightly elliptical uniseriate areolae which are small and regularly spaced, increasing in size from the axial area towards the valve margin and the apices (Fig. 3A–C). Internally, the areolae are occluded by round lattice-structured hymens with short slits around the margin and tiny perforations in the middle (Fig. 4F). SV has a narrow and straight sternum (Fig. 5A, B). In the SEM, it can be seen that, on the SV, the areolae differ greatly between the internal and the external sides (Figs 5C, 6C). Externally, striae are composed of irregular, variably-sized and mostly round areolae, differentiated to the valve edge (Fig. 5A). Areolae are occluded by lattice-structured hymens of marginal slits (Fig. 5C). Internally, the areolae are like puncta. There is a linear and straight axial area (Fig. 6A). Valvocopulae with well-developed digitate fimbriae on both valves are characteristics typical of the *C.crisscrossis*, which are close and heavily silicified fimbriae which seem to be attached to the hyaline rim and are irregularly spaced (Fig. 2A–F). The poles of valvocopulae possess unique ‘sword-shaped’ siliceous extensions that extend towards the interior of the valve and may vary in length (see arrows in Fig. 2A–F).

#### Etymology.

Species was named for its crisscross-shaped central area of the raphe valve.

#### Distribution and ecology.

So far, the new species has been collected at Qingyi River and Maolan Nature Reserve. The Qingyi River population of the new species appears to have a higher abundance in that location than populations from the Maolan Nature Reserve. The habitat of the new species is characterised by circumneutral to alkaline pH (7.5–8.5) and conductivity (120–305 µs/cm) and temperature range 18.0–24.0 °C.

## ﻿Discussion

The morphological differences between species of *Cocconeis* are limited (Stancheva 2022). *Cocconeiscrisscrossis* sp. nov. was compared with other related species (e.g. *C.pediculus* Ehrenberg, *Cocconeischernobyliensis* Genkal, Shcherbak & Semenyuk and *C.molesta* Kützing) which are structurally similar under the light microscope: they all have an elliptical valve outline, concave valve curvature, as well as the size and the arrangement of the striae which are almost parallel in the centre, radiate and slightly curved towards the apices. While the most obvious differences are mainly with respect to the central area under the light microscope. The central area of *C.crisscrossis* sp. nov. extends irregularly to both sides. That feature is absent or not as distinct in morphologically similar taxa. it is one characteristic that distinguishes our new species from other similar species (Table 2).

**Table 2.** Comparison of morphological characteristics of *Cocconeiscrisscrossis* sp. nov. and closely-related taxa.

	*Cocconeiscrisscrossis* sp. nov.	*Cocconeispediculus*	*Cocconeisсhernobyliensis*	*Cocconeismolesta*
Reference	This study	Jahn et al. (2009)	Genkal et al. (2022)	Riaux-Gobin et al. (2016)
Valve outline	Elliptical	Elliptical to somewhat rhombic-elliptical	Elliptical-lanceolate	Elliptical
Length (µm)	12.5–42.0	(11)13.5–40.0(56)	20–36.5	16.4
Width (µm)	9.5–25.0	(6)11.8–26.5(37)	16.5–28.9	9.1
Valvocopulae (RV and SV)	Close, with fimbriate margins, poles have extensions	Close, fimbriate margins in the central part of the valve	Nd	Nd
Central area (RV)	Irregular extends to submarginal to form a cross structure	Small, more or less oval	Narrow, well developed	Narrow, and half a valve in length
Raphe distal endings (RV)	Straight	Straight	Straight	Deflected
Striae (RV)	18–22/10 µm	14–22(24)/10 µm	11–18/10 µm	ca. 30/10 µm
Striae (SV)	18–22/10 µm	14–22(24)/10 µm	12–17/10 µm	40–42/10 µm

The morphology of the RV, the SV and their connecting elements, such as the valvocopulae and the cingulum, are essential for delimiting species within the genus (Riaux-Gobin et al. 2013; Stancheva 2022). The valvocopula system and arrangement show significant variation amongst *Cocconeis* taxa, ranging from intricate fimbriae to no ornamentation; however, the morphology of the valvocopulae is thought to be diagnostic for each taxon (Holmes et al. 1982; Kobayasi and Nagumo 1985; Round et al. 1990; Riaux-Gobin et al. 2021; Stancheva 2022). As shown in Table 2, *C.crisscrossis* was compared with three similar taxa which are structurally different in ultrastructure, mainly in the valvocopulae. *Cocconeiscrisscrossis* and *C.pediculus* possess close valvocopulae with fimbriate margins, while the fimbriae margins of *C.pediculus* are short and present only in the central part of the valve, never at the poles. In contrast to this, valvocopulae of *C.crisscrossis* surround the entire valve, which has longer fimbriae of uneven length and the poles of valvocopulae have distinct ‘sword-shaped’ siliceous extensions towards the interior of the valve, which vary in length, likely owing to different developmental stages. *C.crisscrossis* and *C.pediculus* exhibit remarkable morphological similarity, as both have closed valvocopulae and lack a submarginal hyaline area. These morphological similarities may indicate a close affinity between the two species.

During our observation of this new species, we have discovered that the fimbriate margins of the valvocopula, which occur in the central part of the valve, display considerable morphological stability, while the size and length of the fimbriae exhibit variation. In addition, the size and length of the ‘sword-shaped’ siliceous extensions at the end of valvocopula also vary.

## Supplementary Material

XML Treatment for Cocconeis crisscrossis

## ﻿Additional information

### Author ORCIDs

Pan Yu https://orcid.org/0000-0001-7937-2381

John Patrick Kociolek https://orcid.org/0000-0001-9824-7164

Qingmin You https://orcid.org/0000-0002-5538-8503

### Data availability

All of the data that support the findings of this study are available in the main text.
